# eHealth literacy among hospital health care providers: a systematic review

**DOI:** 10.1186/s12913-023-10103-8

**Published:** 2023-10-24

**Authors:** Gunhild Brørs, Marie Hamilton Larsen, Linn Benjaminsen Hølvold, Astrid K. Wahl

**Affiliations:** 1 Clinic of Cardiology, St. Olavs hospital, Trondheim University Hospital, P.O. box 3250, Torgarden, Trondheim NO-7006 Norway; 2 Lovisenberg Diaconal University College, Lovisenberggatat 15, Oslo, NO-0456 Norway; 3 University Library, Norwegian University of Life Sciences, Universitetstunet 3, Ås, NO-1433 Norway; 4 Department of Health Sciences, University of Oslo, P.O.box 1084, Blindern, Oslo NO-0317 Norway

## Abstract

**Background:**

eHealth literacy is a key concept in the implementation of eHealth resources. However, most eHealth literacy definitions and frameworks are designed from the perceptive of the individual receiving eHealth care, which do not include health care providers’ eHealth literacy or acceptance of delivering eHealth resources.

**Aims:**

To identify existing research on eHealth literacy domains and measurements and identify eHealth literacy scores and associated factors among hospital health care providers.

**Methods:**

This systematic review was reported in accordance with the Preferred Reporting Items for Systematic Reviews and Meta-Analyses (PRISMA) 2020 checklist. A systematic literature search was conducted in MEDLINE, Cinahl, Embase, Scopus, PEDro, AMED and Web of Science. Quantitative studies assessing eHealth literacy with original research, targeting hospital health care providers were included. Three eHealth literacy domains based on the eHealth literacy framework were defined a priori; (1) *Individual eHealth literacy*, (2) *Interaction with the eHealth system*, and (3) *Access to the system*. Pairs of authors independently assessed eligibility, appraised methodological quality and extracted data.

**Results:**

Fourteen publications, of which twelve publications were conducted in non-Western countries, were included. In total, 3,666 health care providers within eleven different professions were included, with nurses being the largest group. Nine of the included studies used the eHealth literacy scale (eHEALS) to measure eHealth literacy, representing the domain of *individual eHealth literacy*. A minority of the studies covered domains such as *interaction with the eHealth system* and *access to the system*. The mean eHEALS score in the studies ranged from 27.8 to 31.7 (8–40), indicating a higher eHealth literacy. One study reported desirable eHealth literacy based on the Digital Health Literacy Instrument. Another study reported a relatively high score on the Staff eHealth literacy questionnaire. eHealth literacy was associated with socio-demographic factors, experience of technology, health behaviour and work-related factors.

**Conclusions:**

Health care providers have good individual eHealth literacy. However, more research is needed on the eHealth literacy domains dependent on *interaction with the eHealth system* and *access to the system*. Furthermore, most studies were conducted in Eastern and Central-Africa, and more research is thus needed in a Western context.

**Trial registration:**

PROSPERO International Prospective Register of Systematic Reviews (CRD42022363039).

## Background

Health literacy is described as the individual’s *knowledge, confidence and comfort to access, understand, appraise, remember and use information about health and health care* [1], and is crucial for enabling health care providers to integrate evidence-based knowledge in their daily practice [2, 3]. Moreover, health literacy is essential for the health care providers` own health and well-being, in addition to those around them (e.g. patients admitted to the hospital) [1].

In the last decade, digital solutions of service provision and working methods have changed health care providers’ competence requirements [4]. With the growing use of digital technology in the last decade, the concept of digital health competency has received considerable attention [4–6], while the concept of electronic health (eHealth) literacy is still unexplored among health care providers.

The concept of eHealth literacy was introduced by Norman and Skinner in 2006, defined as *the individual’s ability to seek, find, understand, and appraise health information from electronic sources and apply the knowledge gained to address or solve a health problem*. The concept was built on the well-known Lily model and measured with the tool eHealth literacy scale (eHEALS) [7]. The Lily model consists of six core literacies forming the basic skills required to optimise individuals’ experiences of eHealth sources [8]. However, as the Lily model was developed for the first generation of eHealth (web 1.0), the skills and confidence in using digital interactions and social media are not part of the model (web 2.0) [9]. Therefore, as advances in technology offer health care providers and patients new ways to interact with and manage such information about health and services, other researchers have continued to develop the concept [10–14]. This has led to two definitions that include possibilities for communication through eHealth sources in all contexts of health care [11, 12]. Furthermore, a new comprehensive framework has been developed. The eHealth literacy framework (eHLF) contains seven scales that provide a new way to understand the interaction and relationship between individuals and the system, in addition to the individual abilities and resources [14]. Moreover, these seven scales are divided into three domains; (1) *Domain dependent on the basic individual eHealth literacy*; (2) *Domain dependent on how the individual interacts with the eHealth system*; and (3) *Domain dependent on the system (e.g. access to hardware or an internet connection when needed)*. The interaction between the individual and the system is where unique aspects of the revised concept of eHealth literacy start to unfold [14]. Unfortunately, most of these definitions and frameworks for eHealth literacy have been designed from the perceptive of the individual receiving eHealth care, and not from that of the health care providers who provided the care by using eHealth resources [12].

In its broadest sense, eHealth is concerned with improving the flow of information, through electronic means, to support delivery of health services and the management of health systems [15]. Despite substantial advances to data and that health care providers having relatively high digital literacy, challenges in health care providers use of eHealth persists [16, 17]. Among those challenges, poor eHealth literacy has been highlighted as a common barrier to the implementation of eHealth resources [18, 19]. That being said, the scientific literature underpinning these barriers for technology integration among health care providers is weak [20], and the concept of eHealth literacy among health care providers almost absent. To our knowledge, two systematic reviews have been published that summarise the digital health competencies among health care providers [5, 6]. One of those identified three studies assessing eHealth literacy among primary health care providers [6]. However, the latter systematic review did not give any attention to eHealth literacy in the discussion section. The most recent systematic review found that the subcategory *Self-rated competencies* containing the concept of eHealth literacy was assessed by five out of 26 studies and all five used the eHEALS which represents the domain dependent on the basic individual eHealth literacy [5]. However, these systematic reviews assessed general digital health competence as an umbrella term, and not eHealth literacy which is a separate established and defined concept. Furthermore, the systematic review did not describe the existing state of eHealth literacy among hospital health care providers, nor associated factors. Thus, due to a number of gaps in the literature, the aims of this systematic review were:

1. To identify existing research on different eHealth literacy measurements and domains among hospital health care providers.
2. To identify eHealth literacy scores among hospital health care providers.
3. To identify factors associated with eHealth literacy among hospital health care providers.

## Methods

### Design

A systematic review with a narrative synthesis was used. The Preferred Reporting Items for Systematic Reviews and Meta-Analyses (PRISMA) guidelines were followed to minimise potential sources of bias [21]. The protocol for this systematic review was registered in the International Prospective Register of Systematic Reviews (PROSPERO) (CRD42022363039) [22].

### Eligibility criteria

The specific eligibility criteria defined a priori are presented in Table 1. Studies that included only a subset of relevant participants were included if they presented the results in subgroups, if not they were excluded.

**Table 1:** Inclusion and exclusion criteria for the studies included in the systematic review

	Inclusion criteria	Exclusion criteria
Participants and setting	Health care providers (e.g. nurses, physicians, physiotherapist, occupational therapist, clinical pharmacists, psychologist) who are working at a hospital (e.g. intensive care unit, general medical ward, outpatient).	Health care providers in primary care (e.g. general practitioner), students at entry-level (e.g. medical students, nursing students), caregivers, post graduating nursing students.
Outcome	The included studies are assessing eHealth literacy as defined a priori; - the ability to seek, find, understand, and appraise health information from electronic sources and apply the knowledge gained to address or solve a health problem. - basic skills to process information, understand health and use eHealth technology. - knowledge about the inner workings of the eHealth systems and having the skills to navigate it. - access to digital services that work and digital services that suit individual needs.	The studies will be excluded it they do not explicit aim to assess eHealth literacy as defined a priori, such as; - assess digital literacy and competence in general, not focusing on digital health solutions in a health context. - assess outcomes not defined as eHealth literacy.
Design	Original research of quantitative design: Cross-sectional design, longitudinal design, cohort design, single arm studies, quantitative results from mixed methods, randomized controlled trials.	Qualitative research, study protocols, different types of reviews, research letters, editorials, case studies, doctoral thesis, conference abstracts.
Language	English, Scandinavian languages.	All other language.

### Search strategy

The systematic search strategy was designed to locate eligible studies published in English and Scandinavian languages. A team of clinical researchers (GB, AW, MHL) and a librarian (LBH) agreed on a search strategy for MEDLINE, which was adapted for use in Cinahl, Embase, Scopus, PEDro, AMED and Web of Science. The timeframe was from inception to November 18th, 2022, we sat no limit on the year of publication, as we wanted to describe the entire range of research relevant for our research questions. The search strategy was made available through DataverseNO [23]. Articles identified through references in the included studies and hand searches were considered for inclusion.

### Data management

**Fig. 1:**
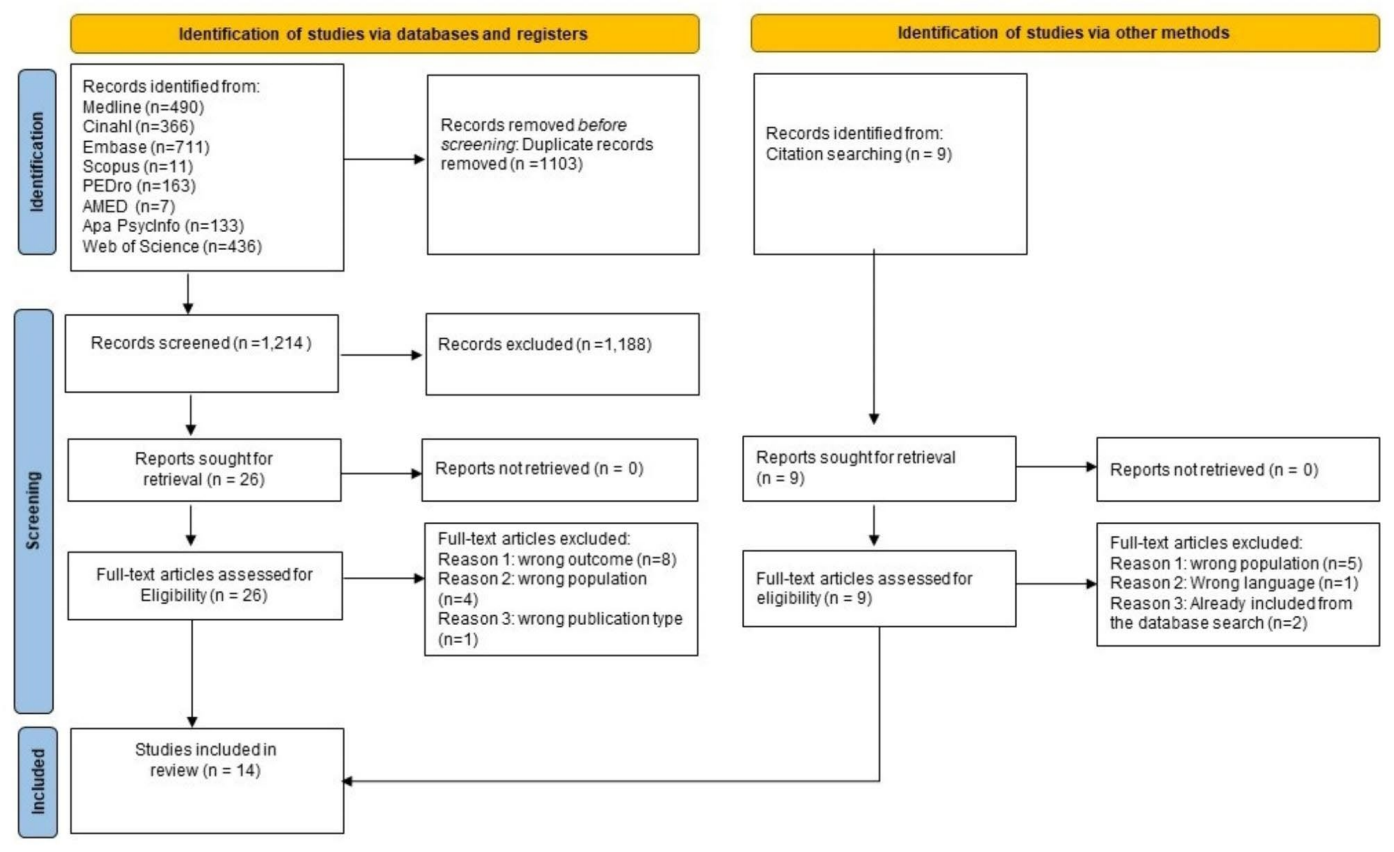
The PRISMA flow diagram [21] describing selection procedure of reviewed articles

The search results from the different electronic databases were combined in a single EndNote library by the librarian (LBH). The librarian identified and removed duplicates. All search results were subsequently uploaded to Rayyan (Rayyan Systems Inc) for storage and facilitation of blinding during the screening process. According to the pre-defined inclusion and exclusion criteria all selected titles and abstracts were scanned independently by two researchers (MHL) and (AKW). The full-text versions of potentially relevant articles were obtained and assessed independently for eligibility by two researchers (MHL and AKW). Any disagreements were resolved through discussion with a third researcher (GB). Three researchers (GB, AKW and MHL) verified the final list of included studies. The reasons for the exclusion of full text publications were recorded using the PRISMA 2020 flow diagram [21]. An overview of the selection procedure of reviewed articles is presented in Fig. 1.

### Data extraction

One researcher (GB) extracted the data to a standardized data collection form that included the following data: year of publication, country of origin, the aims, study design, time period, sample size, health care profession, context, definitions of health literacy, eHealth literacy domains defined a priori, eHealth literacy measures, and findings related to the research questions of the review. A second researcher (AKW) assessed the data extraction for accuracy. Disagreements were resolved through discussion with a third researcher (MHL).

### Quality appraisal

The quality appraisal was systematically assessed using the Joanna Briggs Institute (JBI) critical appraisal checklist for analytical cross-sectional studies [24]. The checklist contains eight questions, however question 3 (Was the exposure measured in a valid and reliable way?) and question 4 (Were objective, standard criteria used for measurement of the condition?) were assessed as Not applicable in the selected publications, as these types of studies did not include any exposures or set out to measure a condition. Hence, we assessed the remaining six questions. Three researchers (GB, MHL and AW) independently conducted the quality appraisal in pairs, and any disagreements were resolved through discussion. The scores applied were “Y” (yes) when the item was satisfied, “N” (no) when the item was not satisfied, and “U” (unclear) when the information contained in the study was not sufficient. To comprehend all studies, their methodological quality was not considered an exclusion criterion.

### Data synthesis and analysis

A descriptive summary of the included publications was performed. To answer the first aim, the eHealth literacy scales used in the included studies were operationalised into three major domains defined a priori; (1) *Domains dependent on the basic individual eHealth literacy*; (2) *Domains dependent on how the individual interacts with the eHealth system*; and (3) *Domains dependent on the system (e.g. access to hardware or an internet connection when needed)*. These three major eHealth literacy domains were based on the eHLF [14]. To answer the second aim, eHealth literacy was described according to the measurement used to assess it. Moreover, a comparison was made between health care professions. To answer the third aim, factors associated with eHealth literacy were categorised into groups according to the phenomena being investigated.

## Results

### Overview

A total of 1,212 publications were identified from the systematic literature search after removal of duplicates. After the first screening, 26 publications were assessed in full text, of which 14 met the inclusion criteria. One study identified from citation searching was assessed as eligible. During the quality appraisal and the data extraction process, one more study was excluded as it did not meet the inclusion criteria for the population. Figure 1 shows the study selection process in the PRISMA 2020 flow diagram [21].

### Characteristics of included studies

**Fig. 2:**
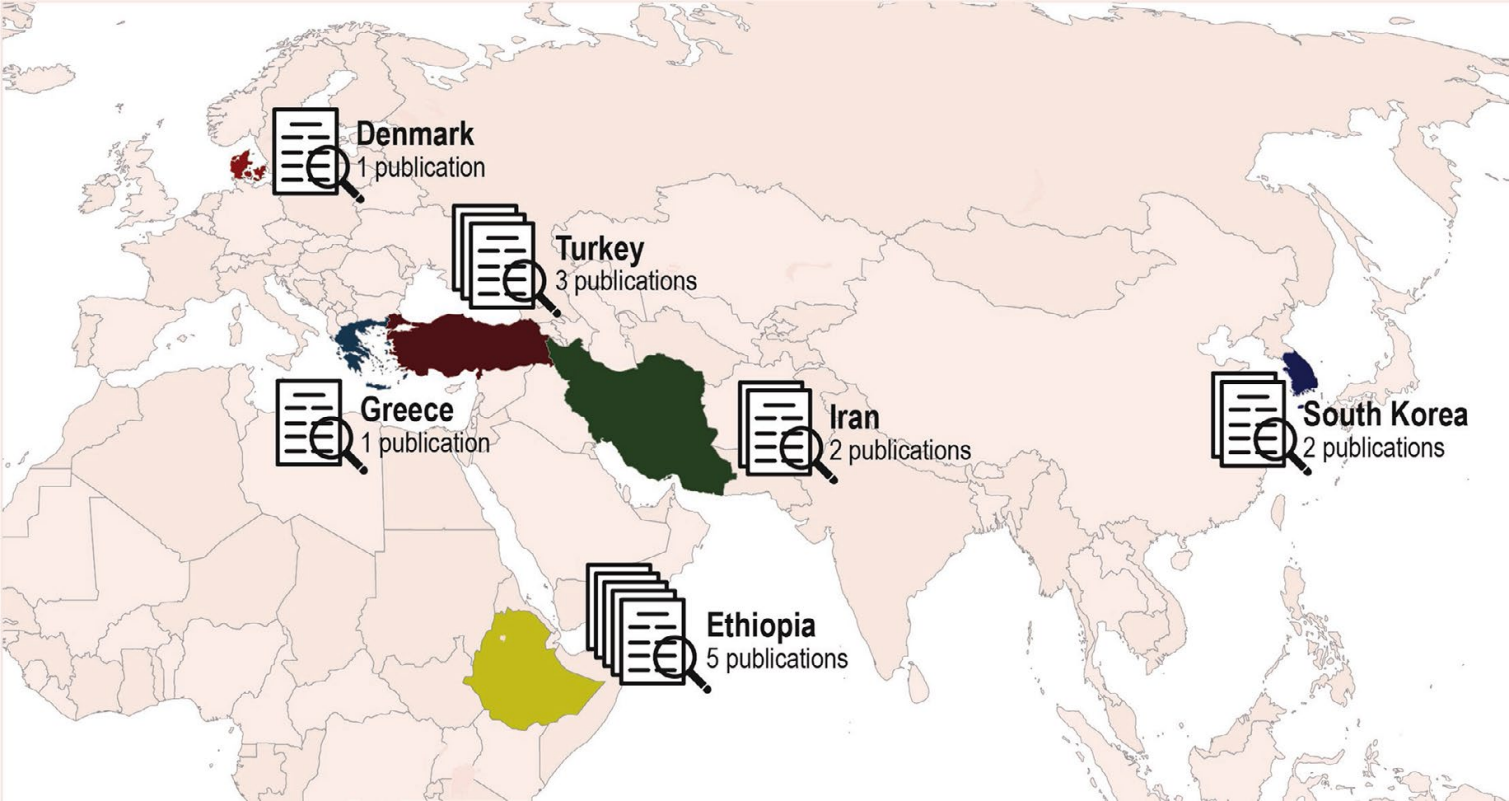
Overview over countries where the studies were conducted

In total, 14 publications from 10 different studies were included. The characteristics of the included studies are presented in Table 2. Thirteen of the publications used a cross-sectional design [25–37], and one used a longitudinal design with two cross-sectional samples [38]. The latter study assessed eHealth literacy before and after the implementation of an integrated electronic health record (HER) system. The other studies included did not assessed eHealth literacy based on a particular type of eHealth source or resource. The publications included were conducted in Ethiopia [27, 33–35], Turkey [25, 36, 37], South Korea [30, 31], Denmark [38], Iran [26, 28] and Greece [32] over a period from 2015 to 2021 (Fig. 2). In total, 3666 health care providers within 11 different professions were included. Nine studies included health care providers from three or more professions (e.g. nurse, physician, midwife, laboratory) [25, 27–29, 33–35, 37, 38]. Two studies included health care providers from two different professions, more precisely nurses and nursing assistants [32], and nurses and physicians [37]. Three studies included nurses as the only health care profession [26, 30, 31, 36]. Hence, approximately two-thirds were nurses.

**Table 2:** Characteristics of studies to be extracted in the systematic review (n = 14)

Author, year, Country of origin	Aims	Study design, time period, and sample size	Health care profession and context	Definition of eHealth literacy	eHealth literacy domains	eHealth literacy measures	Main results related to eHealth literacy and the aims of the review
Ahmed et al., 2022, Ethiopia.	To assess health professionals’ digital health literacy level and associated factors.	Cross-sectional. Data was collected from January to April 2021. n = 401	Nurses (n = 124), physicians (n = 107), midwifes (n = 98), labortorians (n = 49), others (n = 23). Public hospitals in the Illubabor and Buno Bedele zones, Ethiopia.	Yes, a non-established definition.	Domains dependent of the basic individual eHealth literacy.	Digital health literacy skills.	Median eHealth literacy score was 27.4 (SD 8.3), 43.6% had high eHealth literacy. A high eHealth literacy was associated with high computer literacy, higher educational level, higher income, perceived digital tool as useful and easy to use, favourable attitude to eHealth, god knowledge of eHealth, higher frequency internet use.
Alipour and Payandeh, 2022, Iran.	To evaluate and compare the level of digital health literacy of different health care workers.	Cross-sectional, Data was collected in 2021. n = 375 (61%)	Physicians (n = 17), nurses (n = 251), medical records (n = 63), radiology (n = 13), pharmacy (n = 7), laboratory (n = 24). Five teaching hospitals in Iran.	Yes, an established definition.	Domains dependent of the basic individual eHealth literacy and on how the individual interact with the eHealth system.	The Digital Health Literacy Instrument (DHLI).	The healthcare workers have desirable or very desirable literacy in all of the investigated eHealth literacy scales, but relatively far from achieving the very desirable level in the categories related to *determining relevance* and *evaluating reliability*. The mean digital health literacy was significantly different based on level of education, hospital, and job category.
Chereka et al., 2022, Ethiopia.	To assess digital health literacy to share COVID-19 related information and associated factors among healthcare providers.	Cross-sectional. Data was collected from April to May 2021. n = 456 (95.8%)	Doctors (n = 85), Nurses (n = 181), laboratory (n = 91), anesthesia (n = 11), pharmacy (n = 57), radiology (n = 6) COVID-19 treatment center hospitals in the Amhara region, Northwest Ethiopia.	No definition.	Domains dependent of the basic individual eHealth literacy.	Digital health literacy to share COVID-19 related knowledge.	In total, 50.4% of health care providers were at a high level in eHealth literacy to sharing of COVID-19 related information. Higher eHealth literacy was associated with higher education level, access to smartphone, had computer training, favourable attitude towards eHealth, perceived digital tool as useful and ease to use.
Cho et al., 2018, South Korea.	To assess eHealth literacy and health-promoting behaviours among hospital nurses and to determine whether eHealth literacy was associated with their health-promoting behaviours.	Cross-sectional. Data was collected from March to May 2016. n = 485	Nurses (n = 485). Five hospitals in South Korea.	Yes, an established definition.	Domains dependent of the basic individual eHealth literacy.	eHealth literacy scale (eHEALS).	The mean eHealth literacy score was 28.21 (SD 0.38) (8–40). Higher eHealth literacy was associated with better health-promoting behaviour.
Gartrell et al., 2020, South Korea.	To examine the factorial validity of the eHealth Literacy Scale among hospital nurses and to investigate the associations of its components with health promoting behaviours and nursing performance quality.	Cross-sectional. Data was collected from March to May 2016. n = 484 (95%) *same as Cho et al., 2018	Nurses (n = 485) Five hospitals in South Korea. *same population as Cho et al., 2018	Yes, an established definition.	Domains dependent of the basic individual eHealth literacy.	eHealth literacy scale (eHEALS)	Confirmed a 3-factor model (Awareness, Skills and Evaluate). Higher *Awareness*, *Skills* and *Evaluate* were associated with having better health-promoting behaviour.
Isazadeh et al., 2019, Iran	To investigate the electronic health literacy level in nurses working at selected military hospitals in Tehran in 2019.	Cross-sectional, Data was collected in 2019. n = 135	Nurses (n = 135). Three military hospitals in Tehran.	Yes, an established definition.	Domains dependent of the basic individual eHealth literacy.	eHealth literacy scale (eHEALS).	The mean score of the electronic health literacy of nurses was 31.72 (SD 5.51). Nurses’ eHealth literacy was significantly correlated with age, working hospital, and education level.
Kayser et al., 2022, Denmark.	To investigate how a newly developed and modified instrument measuring the medical staff’s eHealth can be used to inform the system provider and the health care organization in the implementation process and evaluate whether the medical staff’s perceptions of the ease of use change and how this may be related to their level of eHealth literacy.	Longitudinal design whit two cross-sectional samples. Data was collected from November 2015 to March 2016. Sample 1: n = 194 (65.8%) Sample 2: 198 (67.1%)	Physicians (n = 46/50) Medical secretary (n = 29/26), Nursing assistants (n = 16/15), nurses (n = 97/104), others (n = 6/3). The Department of Medisin C, Herlev-Gentofte University Hospital.	Yes, an established definition.	Domains dependent of the basic individual eHealth literacy, on how the individual interact with the eHealth system and of the system.	Staff eHealth literacy questionnaire (eHLQ staff).	Staff eHLQ scale 1–3 were at a relatively higher level, but lower at scale 4–7. The physicians scored higher on Staff eHLQ2, and lower on eHLQ6 and eHLQ7 compared to other health care providers. Staff eHLQ scale 1–4 was negative correlated to age. Males had higher score on eHLQ5 compared to female. Staff eHLQ was positive correlated with experience of quick and easy access to information, sharing of data to reduce double registration and stability of IT systems. A small decrease in the staff eHLQ5 at 3-month follow-up.
Kritsotakis et al., 2021, Greece.	To report on eHealth literacy levels in nurses and to explore its associations with the nursing practice environment.	Cross-sectional. Data was collected from February to March 2019. n = 200 (74.34%)	Nurse (n = 121), assistant nurses (n = 79). Tree secondary and one primary general-care hospital in Greece.	Yes, a non-established definition.	Domains dependent of the basic individual eHealth literacy.	eHealth literacy scale (eHEALS).	Mean eHealth literacy score 30.7 (SD 5.8) (8–40). There was no statistical significant difference between the two professions. A higher eHealth literacy was associated with better collegial nurse-physician relationship and nurse participation in hospital affairs.
Şayik and Uçan, 2022, Turkey.	To determine the level of anxiety and eHealth literacy and related factors among physicians and nurses working in adult and/or paediatric inpatient and intensive care facilities where COVID-19 patients were cared for during the pandemic.	Cross-sectional. Data was collected between December 2018 and January 2021. n = 161	Physician (n = 58), nurses (n = 103). Adult and/or paediatric inpatient and intensive care units.	Yes, a non-established definition.	Domains dependent of the basic individual eHealth literacy.	eHealth literacy scale (eHEALS).	The mean eHealth literacy score was 28.72 (SD 7.74) (8–40). There was a statistical significant difference between the mean eHEALS score for physician (30.7) and nurses (27.6). Higher eHealth literacy was associated with being married, higher education, did not think that they needed professional psychological support during the COVID-19 pandemic.
Shiferaw and Mehari, 2019, Ethiopia.	To assess the extent of Internet use and eHealth literacy among health care providers.	Cross-sectional. Data was collected from November 2017 to January 2018. n = 291 (98.6%)	Doctors (n = 19), nurses (n = 88), officers (n = 9), technicians (n = 50), midwifes (n = 64), pharmacist (n = 57). The University of Gondar Comprehensive Specialized Hospital, northwest Ethiopia.	Yes, an established definition.	Domains dependent of the basic individual eHealth literacy.	eHealth literacy scale (eHEALS).	Mean eHealth literacy score 27.84 (SD 5.69) (8–40). Almost 70% reported high eHealth literacy (Cut-off eHEALS score 26) (Medical doctor 100%, nurse 73.9%, health officer 100%, lab technician 70%, midwife: 56.3%, pharmacist 61.4%). The majority with high eHealth literacy were aged 20–29 years and females.
Tesfa et al.,. 2021, Ethiopia.	To assess the level of eHealth-information resource utilization and identify associated factors among health professionals at teaching hospitals in Amhara, Ethiopia.	Cross-sectional. Data was collected from February to May 2020. n = 383	Nurses (n = 158), doctors (n = 94), pharmacy (n = 30), midwifes (n = 54), laboratory (n = 24), others (n = 23). A specialized teaching hospitals in Amhara.	No definition.	Not described.	Not described.	Those with higher eHealth literacy were more likely to use electronic health-information resources compare with those with lower eHealth literacy.
Tesfa et al., 2022, Ethiopia.	To assess the eHealth literacy level and its associated factors among health professionals.	Same as Tesfa et al.,. 2021.	Same population as Tesfa et al.,. 2021.	Yes, an established definition.	Domains dependent of the basic individual eHealth literacy.	eHealth literacy scale (eHEALS).	The mean score for eHealth literacy was 29.21 (SD 7.08) (5–40), and 58.7% had high eHealth literacy. A higher eHealth literacy was associated with better computer access, good computer knowledge, and perceived digital tool as useful and ease to use.
Yoğurtcu and Haney, 2022, Turkey.	To examine and determine the relationship between e-health literacy and the health promoting behaviours.	Cross-sectional. Data was collected from June to august 2019. n = 451	Nurses (n = 451). Two large training and research hospitals in Izmir, Turkey.	Yes, an established definition.	Domains dependent of the basic individual eHealth literacy.	eHealth literacy scale (eHEALS).	Mean eHealth literacy score 29.87 (SD 5.39) (8–40) Higher eHealth literacy were associated with better health promoting behaviours.
Özer et al. 2021 Turkey.	To examine the effects of nurses’ and other healthcare workers’ perceptions of cyberchondria on eHealth literacy.	Cross-sectional, n = 220	Nurses (n = 140), others (n = 80). A public hospital in Burdur, Turkey.	Yes, a non-established definition.	Domains dependent of the basic individual eHealth literacy.	eHealth literacy scale (eHEALS).	Moderate eHealth literacy. Higher eHealth literacy was associated with increased cyberchondria excessiveness dimension.

### eHealth literacy domains

Two publications did not define the concept of eHealth literacy [29, 35], four publications more or less described eHealth literacy without using an established definition [25, 27, 32, 37], and eight used the established definition of eHealth literacy defined by Norman and Skinner in 2006 [7, 8]. Nine of the included publications used the eHealth literacy scale (eHEALS) as a measure of eHealth literacy, which represents domains dependent on the basic individual eHealth literacy [25, 26, 30–34, 36, 37]. One publication used the Digital Health Literacy Instrument (DHLI), which represents the domains dependent on the basic individual eHealth literacy and on how the individual interacts with the eHealth system [28]. One other study used the Staff eHealth literacy questionnaire (Staff eHLQ), which represents the domains dependent on the basic individual eHealth literacy, on how the individual interact with the eHealth system and on the system (e.g. access to hardware or an internet connection when needed) [38]. Two publications did not use a study-specific tool to measure eHealth literacy. However, according to the content of the items, these publications represent domains dependent on the basic individual eHealth literacy [27, 29]. One publication did not specify the tool used to measure eHealth literacy [35].

### eHealth literacy among hospital health care providers

Overall, the mean eHEALS score in the studies ranged from 27.8 to 31.7 (8–40). The studies assessing eHealth literacy among nurses in South Korea [30], Turkey [36] and Iran [26] reported a mean eHEALS score of 28.2, 29.9 and 31.7, respectively. A study from Greece, which included nurses and nursing assistants, reported a mean eHEALS score of 30.7 [32]. A study from Turkey which included nurses and physician reported a mean eHEALS score of 28.7. However, there was a statistically significant difference between the mean eHEALS score for physicians (mean score of 30.7) and nurses (mean score of 27.6) [37]. The studies from Ethiopia, which included three or more health care providers in their sample, reported a mean eHEALS score of 27.8 [33] and 29.2 [34]. The latter studies categorised eHealth literacy as high or low, and reported that 59% [34] and 70% [33] of the health care providers had high eHealth literacy.

The study, which used the Digital Health Literacy Instrument (DHLI) reported that the health care providers have very desirable literacy in the scales *Protecting privacy*, *Operational skills*, *Navigation skills* and *Information searching*. Furthermore, they had desirable level in *Adding content*, *Determining data relevancy* and *Evaluating data reliability*.

The study that used the Staff eHealth literacy questionnaire (Staff eHLQ) reported a relatively high score on the Staff eHLQ scale 1 to 3 (*Using technology to process health information*, *Understanding of health concepts and language*, and *Ability to actively engage with digital services*), while they reported a lower score on the Staff eHLQ scale 4 to 7 (*Feel safe and in control*, *Motivated to engage with digital services*, *Access to digital services that work*, and *Digital services that suit individual needs*). Physicians scored higher on Staff eHLQ2 (*Understanding of health concepts and language)*, but lower on Staff eHLQ scale 6 and eHLQ scale 7 (*Access to digital services that work*, and *Digital services that suit individual needs)* compared to medical secretaries, nursing assistants and nurses.

Two studies assessed eHealth literacy in relation to COVID-19 by using a questionnaire tailored for this purpose (e.g. *I know how to use the internet to answer my questions about the COVID-19 pandemic*). They reported that 40 to 50% of health care providers had a higher eHealth literacy score [27, 29].

### Factors associated with eHealth literacy

A higher score on the eHEALS was associated with demographic characteristics such as age, being married and having higher education [26, 37]. Moreover, a higher score on the eHEALS was associated with better health promoting behaviour [30, 31, 36], and work-related factors, such as a better collegial nurse-physician relationship and nurse participation in hospital affairs [32]. Those with a higher eHEALS score had better computer access, and knowledge, and perceived digital tools as useful and ease to use [34, 35]. Finally, the perception of cyberchondria explained 12% of the total variance in eHealth literacy [25].

The mean eHealth literacy measured using DHLI was significantly different based on health care providers’ level of education, hospital affiliation and job category [28]. The study that used the Staff eHLQ reported that males had a higher score on the eHLQ scale 5 (*Motivated to engage with digital services*) compared to females. Moreover, the Staff eHLQ scale 1–4 (*Using technology to process health information*, *Understanding of health concepts and language*, *Ability to actively engage with digital services, and Feel safe and in control*) was negatively correlated with age. Finally, the Staff eHLQ was positive correlated with experience of quick and easy access to information, sharing of data to reduce double registration and stability of IT systems [38].

The studies assessing eHealth literacy in relation to COVID-19 reported that higher eHealth literacy was associated with a higher educational level, higher income, access to a smartphone, high computer literacy and perception of digital tools as useful and easy to use. These participants also had a favourable attitude to eHealth, good knowledge of eHealth and a higher frequency of internet use [27, 29].

### Quality appraisal

To enhance trustworthiness, all studies were quality appraised using the JBI critical appraisal checklist for analytical cross-sectional studies. Overall, on average, the studies reported an adequate score (Yes) in four of the included six questions, however with some variety. One study reported an adequate score in six out of six questions [30], and one study reported an adequate score in only two out of six questions [35]. Some questions showed quality problems; the criteria for inclusion (question 1) were sufficiently defined in only half of the included articles and confounding factors (question 5) were clearly identified in four articles, while strategies to deal with confounding factors were stated in five of the 14 articles (question 6). On a positive note, the study subjects were described in 12 of the 14 included articles (question 2), the outcomes were measured in a valid and reliable way in 11 (question 7), and appropriate statistical analysis was used in all articles (question 8) (Table 3).

**Table 3:** The Johanna Briggs Institute critical appraisal for analytical cross-sectional studies

	Q1	Q2	Q3	Q4	Q5	Q6	Q7	Q8	Yes	Unclear	No
Ahmed et al., 2022	Yes	Yes	NA	NA	Yes	Yes	No	Yes	5	0	1
Alipour and Payandeh, 2022	Unclear	Yes	NA	NA	Unclear	Yes	Yes	Yes	4	2	0
Chereka et al., 2022	Unclear	Yes	NA	NA	Yes	Yes	Yes	Yes	5	1	0
Cho et al., 2018	Yes	Yes	NA	NA	Yes	Yes	Yes	Yes	6	0	0
Gartell et al., 2020	No	Yes	NA	NA	Unclear	Unclear	Yes	Yes	3	2	1
Isazadeh et al., 2019	Yes	Unclear	NA	NA	No	No	Yes	Yes	3	2	1
Kayser et al., 2022	Yes	Yes	NA	NA	No	No	Yes	Yes	4	2	0
Kritsotakis et al., 2021	Unclear	No	NA	NA	Yes	Yes	Yes	Yes	4	1	1
Şayik and Uçan, 2022	Yes	Yes	NA	NA	N0	No	Yes	Yes	4	0	2
Shiferaw and Mehari, 2019	Yes	Yes	NA	NA	No	No	Yes	Yes	4	0	2
Tesfa et al., 2021	No	Yes	NA	NA	No	No	Unclear	Yes	2	1	3
Tesfa et al., 2022	Unclear	Yes	NA	NA	No	No	Yes	Yes	3	1	2
Yoğurtcu and Haney, 2022	Yes	Yes	NA	NA	No	No	Yes	Yes	4	0	2
Özer et al., 2021	Yes	Yes	NA	NA	No	No	Yes	Yes	4	0	2

NA = Not applicable

Q1: Were the criteria for inclusion in the sample clearly defined?

Q2: Were the study subjects and the setting described in detail?

Q3: Was the exposure measured in a valid and reliable way?

Q4: Were objective, standard criteria used for measurement of the condition?

Q5: Were confounding factors identified?

Q6: Were strategies to deal with confounding factors stated?

Q7: Were the outcomes measured in a valid and reliable way?

Q8: Was appropriate statistical analysis used?

## Discussion

Only half of the studies presented an established definition of eHealth literacy, which is important for how the concept is operationalised and measured (e.g. construct validity). The findings showed that health care providers have good individual eHealth literacy, primarily measured with the first-generation patient reported outcome measurement for eHealth literacy (the eHEALS) representing the domains dependent on the basic individual eHealth literacy. Assessments on the eHealth literacy domain dependent on how the individual interacts with the eHealth system and domain dependent the system itself (e.g. access to hardware that work) were almost absent. Moreover, the studies were conducted in six different countries, primarily non-Western countries. This, as well as the low number of studies being published on this topic in the era of eHealth development, indicate that eHealth literacy among hospital health care providers is underexplored globally, specifically in Western countries. This is important as eHealth literacy is a key to seeking health information online for appropriate decision-making [39]. Therefore, eHealth literacy resources must be available to support hospital health care providers to access, remember, understand and use up-to-date evidence-based knowledge and help the patients to take health-related decisions through eHealth sources in their daily practice. Increased knowledge about factors that increase eHealth literacy can guide better health care practice and improve patient safety in the era of eHealth. Therefore, to achieve equality in health care service in the era of eHealth, the national government needs to place eHealth literacy among hospital health care providers on the agenda.

As the eHEALS is based on the Lily model, which was developed for the first generation of eHealth back in 2006, these publications may not provide enough knowledge important to implementing eHealth resources that enable digital interactions with patients [12, 14]. Approximately seven eHealth literacy instruments were available in 2021 [40]. Among those, the DHLI and the eHLQ (used in two publications included in this systematic review [28, 38]) were described as second-generation measurements with a broader scope suitable for individuals living in the web 2.0 era of eHealth [40]. However, these measurements were only available in a few languages. The eHEALS has been translated and assessed for psychometric properties in at least 17 languages [40]. Thus, one reason for the large amounts of publications using the eHEALS may be that the second-generation eHealth literacy measurements have not been available in the language in which the studies were carried out.

Furthermore, a discrepancy was identified between the definitions and measurements used in very many of the publications. A previous systematic review has also reported this unfortunate absence of established eHealth literacy definitions [40]. Additionally, a discrepancy between definitions and measurements has also been reported for the concept of health literacy [41]. Defining the concept to be measured is the most basic and important starting point, as it determines the scope of the instrument being developed and will impact on measurement properties [40]. Therefore, it is relevant to question the content and construct validity, and responsiveness of the measurements used to assess eHealth literacy among hospital health care providers. These findings highlight the importance of developing new definitions that conceptualise eHealth literacy among health care providers and operationalise the concept into new and broader measurements. This will secure more reliable and valid assessments of eHealth literacy among health care providers, which can advance the research field. However, the Staff eHLQ is a modified version of the eHLQ. By changing the perspective of the respondent from themselves to their interaction with patients, this measurement should become more adapted to the health care providers who deliver the eHealth resources, and it is the first questionnaire with this approach. However, further evidence is needed on the psychometric properties of the measurement [38].

The hospital health care providers reported an eHEALS score of between 27.8 and 30.7 (8–40) which represents a high self-perceived eHealth literacy, compared with eHEALS scores reported by younger adults in South Korea who are active online users (eHEALS score 28.06) [42] and by a general Dutch population (eHEALS score 27.6) [43]. Moreover, findings in this current systematic review showed that eHealth literacy measured using eHEALS is associated with health promoting behaviour in the studies conducted in Turkey and South Korea [30, 31, 36]. This is also shown in a previous systematic review on eHealth literacy within different age groups (from teenagers to older adults) and among different populations regardless of disease status [44]. This is interesting as health care providers play an important role in encouraging adherence to public health guidelines [45]. Additionally, non-adherence to lifestyle-related health guidelines among nurses in Scotland and England is reported to be high, which raises concerns about the effectiveness of health promotion during patient interactions [45]. The level of eHealth literacy is one of the main factors in combating too much information including false or misleading information in eHealth sources (e.g. infodemic during the COVID-19 pandemic). Increasing eHealth literacy among health care providers could help to avoid the negative consequences of false or misleading information in daily decision-making [39]. However, further investigation is needed to better understand this association as eHealth interventions will increasingly be used in the future to change negative health behaviour and to promote a healthy lifestyle [46].

Interestingly, eHealth literacy was higher among physicians, than other health care providers. This could be related to physicians’ prior digital training and higher level of education, which may be associated with higher eHealth literacy [38]. These results support previous research reporting that health care providers with higher level of education are more willing to improve their digital health competence [4, 47]. This indicate that hospital health care providers’ eHealth literacy strengths and limitations should be considered when developing eHealth resources. Moreover, to implement measures that respond to the health care providers’ eHealth literacy to improve the health care service in the era of eHealth.

It is a paradox that the countries that have measured eHealth literacy among hospital health care providers are those assumed to have lower awareness and utilisation of eHealth technology, such as Ethiopia, and not the countries that have implemented eHealth on a larger scale. Importantly, hospital health care providers in Ethiopia with a higher eHealth literacy score also had better computer access and knowledge, and perceived digital tools as useful and easy to use [27, 29, 34, 35]. Furthermore, as World Health Organization stated that eHealth has been implemented in the absence of a careful examination of the evidence base [48, 49], inadequate knowledge of health care providers’ eHealth literacy may be one of the reasons why Western countries have faced barriers in the implementation processes. Thus, knowledge of health care providers’ eHealth literacy seems highly relevant for countries with lower economic performance, such as Iran and Ethiopia, when developing eHealth programmes to improve the delivery of health care services [50]. However, this knowledge cannot be generalised to Western countries, where eHealth infrastructure exists to very different extent compared to non-Western countries. Moreover, the pattern in eHealth access, use and engagement is reported to vary across populations in Europe, and tends to be more widespread in urban areas, and less so among people from ethnic minorities and those facing language barriers [51]. This means that the eHealth literacy of health care providers may vary across continents, countries and regions, and may therefore be addressed differently.

### Strengths and limitations

The strength of our systematic mixed studies review lies in its methodological rigour. A systematic approach to collecting data, including a broad search strategy in six databases, developed in close collaboration with an experienced research librarian. The PRISMA guidelines were used to minimise potential sources of bias. In addition, study selection, quality assessment and data extraction were conducted systematically and in parallel by two independent researchers. One limitation could be that the current systematic review only includes quantitative studies, and qualitative studies might have added valuable knowledge to the field.

Furthermore, the measurements used are based on Western conceptualization of eHealth literacy and operationalisation, which may not harmonise with the worldviews of all participants in the studies using these instruments [52]. Therefore, any generalisation of eHealth literacy must be made with caution as context is crucial and factors as described above can influence the results and lead to bias. Our systematic review also had some language restrictions as studies may have been published in other languages that we were not able to identify. A further limitation is the lack of doing meta-analysis to explore the pooled associations between the studies. This was however considered to have limited utility as the included studies were observed to be heterogeneous in execution and choice of outcome measures. The choice of reporting results from studies independent of their quality assessment may also be seen as a limitation.

## Conclusions

The result from this systematic review shows that health care providers have good individual eHealth literacy. However, more research is needed on the eHealth literacy domains dependent on how the individual interacts with the eHealth system and on the system itself, using more comprehensive measures. Studies are also needed to measure multidisciplinary professions’ eHealth literacy in the hospital context. Furthermore, most of the studies were conducted in Eastern and Central-Africa, and more research of higher methodological quality is needed in a Western context.

## Data Availability

The search strategy is available through DataverseNO.

## Abbreviations

DHLI: The Digital Health Literacy Instrument
eHealth: Electronic health
eHEALS: eHealth literacy scale
PRISMA: The Preferred Reporting Items for Systematic Reviews and Meta-Analyses
PROSPERO: The International Prospective Register of Systematic Reviews
Staff eHLQ: Staff eHealth literacy questionnaire
